# Development of Sensitive and Rapid RNA Transcription-based Isothermal Amplification Method for Detection of *Mycobacterium tuberculosis*

**Published:** 2019

**Authors:** Reihaneh Ramezani, Mahdi Forouzandeh Moghadam, Mohammad Javad Rasaee

**Affiliations:** 1. Department of Biomedical Sciences, Women Research Center, Alzahra University, Tehran, Iran; 2. Department of Medical Biotechnology, Faculty of Medical Sciences, Tarbiat Modares University, Tehran, Iran

**Keywords:** Microbial viability, *Mycobacterium tuberculosis*, NASBA-ELISA

## Abstract

**Background:**

The accurate and early diagnosis of tuberculosis is important for its effective management. During the last decade, several molecular methods for detection of Tuberculosis (TB) have been developed. Since RNA especially mRNA has a generally much shorter half-life than DNA, its detection may be useful for the assessment of viability of bacteria. This research is a Nucleic Acid Sequence Based Amplification-Enzyme Linked Immunosorbent Assay (NASBA-ELISA) which was designed and developed for rapid detection of viable *Mycobacterium tuberculosis (M. tuberculosis)*.

**Methods:**

Oligonucleotide primers targeting *tuf* gene encoding viability marker EF-Tu mRNAs were selected and used for the amplification of mycobacterial RNA by the isothermal NASBA Digoxigenin (DIG) labeling process and incorporated with DIG-UTP, reverse transcriptase and T7 RNA polymerase.

**Results:**

Using the NASBA-ELISA system, as little as 17.5 *pg* of RNA of *M. tuberculosis* was detected within 4 *hr* and no interference was encountered in the amplification and detection of viable *M. tuberculosis* in the presence of non-target RNA or DNA. Results obtained from the clinical specimens showed 97 and 75% of sensitivity and specificity, respectively.

**Conclusion:**

The NASBA-ELISA system offers several advantages in terms of sensitivity, rapidity and simplicity for detection of *M. tuberculosis*. Furthermore, due to its simplicity and high sensitivity feature, it could be used in limited access laboratories in a cost-effective manner.

## Introduction

Tuberculosis (TB) is second only to HIV/AIDS as the greatest killer worldwide due to a single infectious agent. In 2016, 10.4 million people fell ill with TB and 1.3 million died from the disease. Over 95% of TB deaths occur in low- and middle-income countries, and it is among the top 5 causes of death for women aged 15 to 44 1–4.

Conventional methods used in developing countries for the diagnosis of TB have certain disadvantages; for example, sputum smear microscopy which is a major technique, is neither sensitive nor specific (requires 10000 to 100000 *organism/ml* for detection) and cannot distinguish viable from non-viable bacilli 5,6. Because of the slow generation time of *Mycobacterium tuberculosis (M. tuberculosis)*, culture is very time-consuming (requires 6 to 8 weeks) and cannot be suitable for early diagnosis and monitoring of therapeutic efficacy. The increase of tuberculosis cases in many countries and the emergence of multidrug-resistantstrains require more rapid and accurate methods to be used in medical laboratories 7,8. Nucleic acid amplification methods reduce the time for diagnosis from weeks to hours, while sensitivity and specificity of these techniques are more reliable than classical methods 8,9.

*M. tuberculosis* DNA is very stable and has been shown to persist in sputum in certain patients even after tuberculosis is cured. However, mRNA has a typical half-life of only a few minutes and the presence of mRNA is a good indicator of bacterial viability 1,10. The most commonly used amplification techniques for detecting mRNA are Reverse Transcriptase PCR (RT-PCR) and Nucleic Acid Sequence-Based Amplification (NASBA) 11,12.

**Figure 1.**
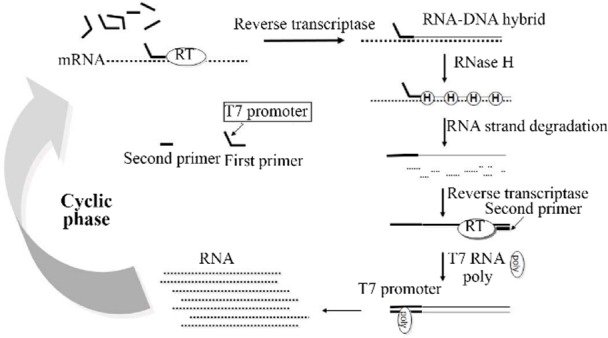
Scheme for the amplification of RNA by the NASBA reaction.

NASBA is a transcriptional based amplification reaction that utilizes three different enzymes to mimic re-troviral replication system specially, Avian Myeloblastosis Virus reverse-transcriptase (AMV), ribonuclease H (RNaseH) and T7 RNA polymerase and they act together to amplify an RNA target, in an isothermal condition (Figure 1) 13,14.

NASBA has several advantages over other mRNA amplification methods; it is an isothermal reaction which obviates the need for a thermal cycler. A single-strand antisense RNA product is produced during NA-SBA, which can be directly hybridized by a probe sequence to accelerate post-amplification interrogation of the product. Another important point is in sample preparation and RNA targets cannot be specially analyzed by RT-PCR in a background of contaminating DNA. To overcome this, DNA contamination must be removed by DNase. In contrast, background DNA does not interfere with the NASBA reaction, as single-stranded RNA is specifically targeted; thus, use of NABA removes the necessity for very specific RNA extraction process 15.

**Figure 2.**
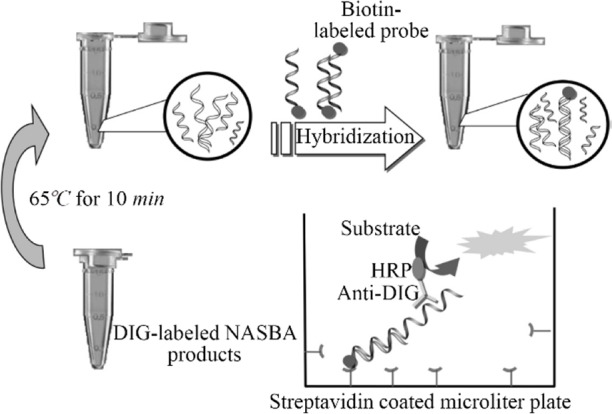
Scheme of ELISA system for detection of DIG-labeled NASBA product.

The aim of the present study was to develop and exploit the RNA transcription-based isothermal amplification method, NASBA-ELISA assay, as an improvement over traditional assays to detect *M. tuberculosis*. In this study, for the first time, NASBA-ELISA technique (Figures 1 and 2) was applied to detect the EF-Tu mRNA as a viability marker in *M. tuberculosis*.

## Materials and Methods

### Bacterial strains

The *M. tuberculosis* (RIVM), H37Rv strain was provided by Department of Tuberculosis, Razi Vaccine and Serum Research Institute, Karaj, Iran.

*Mycobacterium bovis (M. bovis)*, AN5 (ATCC357 26) was also provided by Razi Institute. *M. bovis* (ATCC35726), *Escherichia coli (E. coli)* (ATCC1177 5), *Enterococcus faecalis (E. faecalis)* (ATCC 29212), and *Klebsiella pneumoniae (K. pneumonia)* (ATCC 700603) were used to evaluate the specificity of the NASBA reaction and grown in trypticase soy broth. *M. tuberculosis* H37Rv and strains isolated from patients in Iran were grown in 7H9 broth medium. All these strains were identified by conventional methods and characterized as *M. tuberculosis.*

### Clinical specimens

The 35 sputum samples of patients were collected from Zarifi Laboratory and analyzed as they were received. All specimens were decontaminated by the N-acetylcysteine-NaOH procedure and cultivated in Lowenstein-Jensen Media (7H9 broth medium).

### RNA extraction

After two weeks of culturing of H37Rv strain and patients specimens in 7H9 broth medium and adjusting the turbidity of broth medium, in compliance with no.4 McFarland standard (Barium Sulfate suspension=1.2× 109 *cell/ml*), RNA extraction was performed with the following protocol:

About 5 *ml* of specimen cultures were centrifuged at 5000 *g* for 5 *min* at 4*°C*. Supernatant was decanted and the remaining media were carefully removed. Precipitations were dissolved in TE buffer (1 *mM* EDTA and 10 *mM* Tris-HCl) and mixed by pipetting. Then, they were centrifuged at 5000 *g* for 5 *min* at 4*°C* and precipitations dissolved in lysis buffer (RLT buffer in Qiagen RNasy Mini kit) were recovered and vortexed for 10 *min* with glass beads included (dropped) in the solution. During this step, heating (85*°C*) was used to increase the efficacy of lysis buffer because of *M. tuberculosis* cell wall. After lysing cell wall, the extraction of RNA continued according to the Qiagen RNasy Mini kit. The extracted RNA was stored at 80*°C* until it was used for concurrent amplifications by NASBA.

### DNA oligonucleotides

The primers and probes used in this study are listed in table 1. Tuf18 primer and tuf15 primers were chosen from a highly conserved region of the nucleotide sequence of EF-Tu mRNA *M. tuberculosis* and obtained United States Patent with number of US6489110 16. These sequences were chosen by clustal alignments of variable regions of different microorganism with Meg Align software program. These primers can amplify approximately 203 nucleotides of EF-Tu mRNA.

**Table 1.** The sequence of primers and probe used in this study

**Primer/probe**	**Position**	**Sequence (5′→ 3′)**
TUF15 + T_7_^*^	1057–1039	AATTCTAATACGACTCACTATAGGGAGAAGCTTGGTCGTCGTCGATGGGCGA
TUF18	875–855	CCTCTG TCGAGGAACTGATGA
TUF26	1014–1031	Biotin-ACGAGGAAGTTGAGATCG

* The sequence of T _7_ promoter is underlined.

### Digoxigenin labeling NASBA reaction

NASBA reactions were carried out in a final volume of 25 *μl* containing 12 *mM* MgCl_2_, 12 unit RNase inhibitor (Thermo Scientific), 40 *mM* Tris-HCl, 10 *mM* NaCl, 5 *mM* DTT, 5% DMSO, 0.1 *μg/μl* BSA (Bovine Serum Albumin, Sigma), 1 *mM* ATP, 1 *mM* CTP, 1 *mM* GTP, 0.91 *mM* UTP, 0.09 *mM* Dig-11-UTP, 1 *mM* dNTP, 8 unit M-Mulv RT (Thermo Scientific), 40 unit T7 RNA polymerase (Thermo Scientific), 0.2 *μM* of each primer (tuf18, tuf15) and 5 *μl* of extracted RNA. Primers were provided by MWG Biotech Company (Germany) in dried form and were reconstituted before assembling the reaction. Prior to the addition of enzymes, the reaction mixture was heated at 65*°C* for 5 *min* to destabilize secondary structures of RNA, and it was then cooled to 38*°C* for 1 *min*, for primer annealing and protection of enzymes. Following the addition of enzymes, the reaction mixture was incubated at 38*°C* for 90 *min*, and then the NASBA products were stored at −80*°C* or were used in detection methods.

### Detection method

***Coupling and hybridization protocol for streptavidin coated microtiter plate:*** The digoxigenin labeled NASBA products were detected in solution hybridization process (Enzyme-linked immunosorbent assay). Nunc Immobilizer TM sterptavidin plates/strips for hybridization reaction were used in this study. First, the strips were treated with 300 *μl* 5×SSC buffer (750 *mM* NaCl and 75 *mM* sodium citrate) containing 0.05% (*v/v*) Tween 20. The second structure of NASBA products (ssRNA) was removed by heating at 65*°C* for 10 *min*; Next, a solution of biotinylated probe (tuf26) and 100 *μl* hybridization buffer (50 *mM* sodium-phosphate buffer, pH=7.0) and 10 *μl* of NASBA products was prepared. This solution was then added to the treated wells of Nunc Immobilizer TM sterptavidin plates (100 *μl/well*). The plates were incubated with gentle agitation for 1 *hr* at 38*°C*. The wells were aspirated and washed with PBST, pH=7.2 [Phosphate Buffered Saline containing 0.05% (V/V) Tween 20].

### Colorimetric assay

HRP-Anti DIG (Horse Radish Peroxidase-Anti Digoxigenin) diluted 1:1000 in PBST buffer was added to strips (100 *μl/well*). The strips were incubated with gentle agitation at room temperature for 30 *min*. Then, the strips were aspirated and washed three times with PBST buffer (3×300 *μl/well*). In the final step, 100 *ml* of 2,2-azinodi-(3-ethylbenz-thiazolinsulfonate) substrate (ABTS, Roche) was added to each well and incubated at 37*°C* for 20 *min*.

## Results

Combing the NASBA technique with ELISA system has made it possible to identify the RNA target molecules in a cell with a high sensitivity and precision.

### Optimization of the conditions of NASBA reaction

The different factors effective in NASBA reaction were examined one by one and finally it became obvious that the presence of DMSO in the reaction would increase the specificity of primer annealing to template thus reducing nonspecific amplification (Data is not provided here). By varying the concentrations of NaCl and KCl, different results were obtained, as the concentration of these salts increased, higher concentration of RNA was obtained. However, in very high concentration, the effect was inverse and the amount of product decreased (data is not provided here).

**Figure 3.**
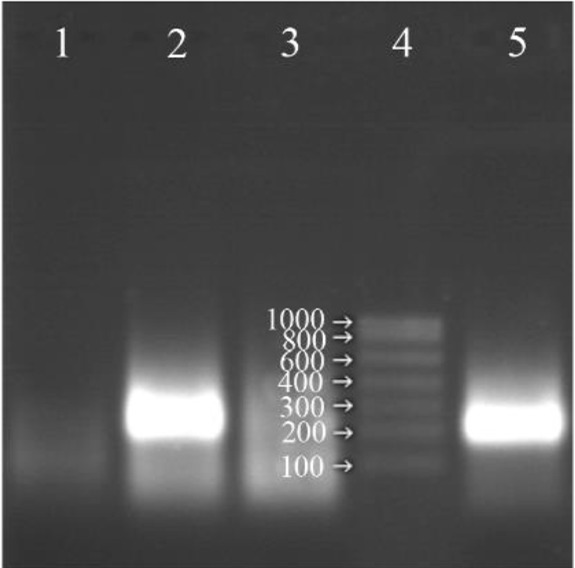
Analysis of the NASBA products (220 *bp*) on 2% agarose gel without using RNase H in the reaction, Lane 1: negative control, Lane 2: NASBA product with RNase H in the reaction, Lane 3: NASBA product in the presence of RNase A, Lane 4: RNA ladder, Lane 5: NASBA product without RNase H in the reaction.

Since most of the reverse transcriptases have RNase H property, the reaction was performed in the absence of RNase H and almost the same intensity of the amplification of RNA on the gel was observed. In other words, RNase H enzyme could be omitted from the reaction and the rest of the procedure could be continued with only two enzymes (Figure 3).

### Labeling NASBA products with digoxigenin

**Figure 4.**
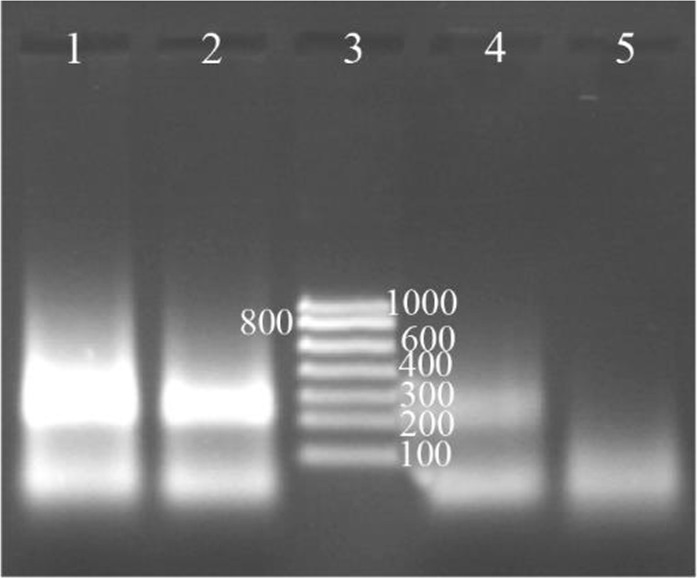
Determination of the digoxigenin labeled NASBA products on 2% gel electrophoresis, Lane 1: NASBA product with 0.09 *mM* digoxigenin, Lane 2: NASBA product with 0.18 *mM* digoxigenin, Lane 3: RNA ladder, Lane 4: NASBA product with 0.35 *mM* digoxin-genin, Lane 5: negative control.

It was formerly assumed that maybe the use of ribonucleotides labeled with digoxigenin (DIG-11-UTP) could affect the performance of reverse transcriptase and T7 RNA polymerase, and decrease the activity of these enzymes. But using this system was successful and NASBA products were labeled easily with digoxigenin. The increase in concentration ratio of Dig-11-UTP to other NTPs leads to a decrease in reaction products. The best results were achieved when 0.09 *mM* of DIG-11-UTP and 0.91 *mM* of UTP were used in the reaction (Figure 4).

### Detection of digoxigenin labeled NASBA products

The detection system is the Enzyme Linked Immunosorbent Assay (ELISA). The ELISA system is a kind of solution hybridization. The specificity of the technique because of the use of specific probes for hybridization is considerably higher than gel electrophoresis method.

Results obtained from the clinical specimens showed 97 and 75% sensitivity and specificity, respectively for NASBA-ELISA assay. The 35 sputum samples of patients were collected from Zarifi Laboratory and analyzed with NASBA-ELISA and bacterial culture. 33 samples were positive with NASBA-ELISA and bacterial culture, and 3 samples were negative by both methods. Only one of the samples had positive culture result, but the NASBA-ELISA technique was not able to detect *M. tuberculosis* in it (Table 2).

**Table 2.** Comparison of culture results with those of the NASBA-ELISA

**Test and result**	**No. of culture results**	

	**Positive**	**Negative**
**NASBA-ELISA**		
**Positive**	33	0
**Negative**	1	3

### Sensitivity of NASBA-ELISA technique

**Figure 5.**
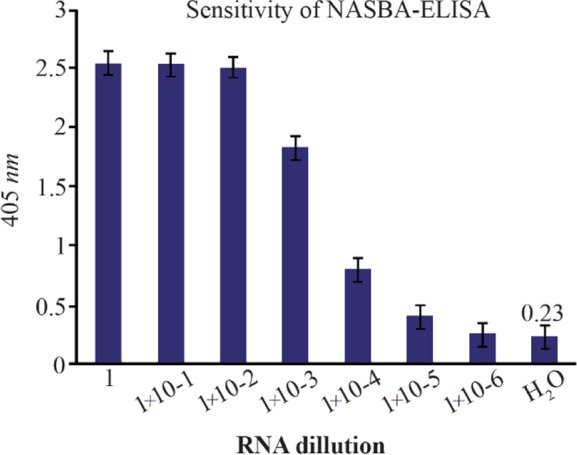
The assay of the sensitivity of NASBA-ELISA DIG-detection system for molecular detection of *M. tuberculosis* EF-Tu mRNA in broth media. Ten-fold serial dilutions of the RNA were used in DIG-detection NASBA-ELISA system. Each dilution was analyzed in triplicate (The concentration of the first dilution is 7 *ng/μl*).

The sensitivity of the NASBA-ELISA was investigated with *M. tuberculosis* (H37Rv) as a model system. This was done by adding a 10-fold serial dilution of purified RNA derived from H37Rv in amounts ranging from 1750 *ng* to 17.5 *pg* to the reactions. A 20 *pg* amount of mRNA could be amplified reproducibly to give a clear signal on ELISA system (Figure 5).

### Specificity of NASBA-ELISA technique

**Figure 6.**
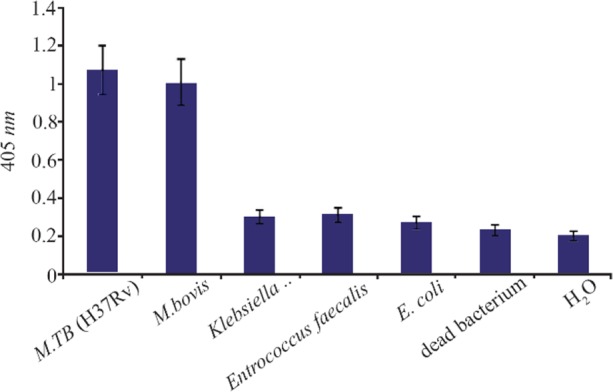
The assay of specificity of NASBA-ELISA DIG- detection system. NASBA-ELISA was performed on non-target RNA samples extracted from *E. coli, K. pneumonia, E. faecalis* and *M. bovis*. NASBA-ELISA was performed on *M. tuberculosis* (H37Rv) as a positive control and H_2_O and dead *M. tuberculosis* as a negative control. The plotted results are averages of triplication analysis.

*M. bovis* (ATCC35726), *E. coli* (ATCC11775), *E. faecalis* (ATCC29212), *K. pneumonia* (ATCC700603) were used to evaluate the specificity of the NASBA reaction and grown in trypticase soy broth. Except *M. bovis*, the results of NASBA-ELISA for the other bacteria were negative and it indicates the specificity of the technique in identifying *M. tuberculosis* (Figure 6). Of course, the differentiation of *M. bovis* from *M. tuberculosis* is very difficult because of the very high genomic similarity between them, which is why the separation of these two strains with the NASBA-ELISA technique could not be successful.

### The function of NASBA technique on dead bacteria

**Figure 7.**
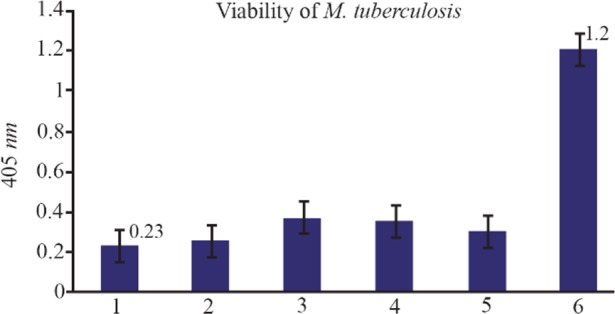
The results of NASBA-ELISA technique on dead and viable micro-organisms: 2, 3, 4 and 5 are related to dead *M. tuberculosis* with high temperature and number 6 to the result of NASBA-ELISA on 10^5^ CFU *ml*^−1^ of viable *M. tuberculosis* grown in broth media. 1 is related to the result of NASBA-ELISA reaction with H_2_O instead of RNA.

The dead bacteria were obtained using high temperatures (95*°C*) for 15–20 *min* and nucleic acid extraction was done and subsequently the NASBA reaction. As shown in figure 7, there exists no amplification to be the reason for nonexistence of EF-Tu mRNA in bacteria cell and certain death of the bacteria.

## Discussion

The accurate and timely diagnosis of tuberculosis is one of the important matters in effective treatment management of this disease. Because *M. tuberculosis* is growing slowly in culture environment (doubling time for H37Rv is 79±5 *hr*) 17, and there are serious limitations in common methods like microscopic examination, which is used to identify this bacterium, the dire need for fast, sensitive and accurate methods to identify *M. tuberculosis* becomes apparent. In the previous decade, several molecule molecular methods have been developed to diagnose tuberculosis 18,19. Among these methods, nucleic acid isothermal amplification methods have been remarkable.

The aim of this study was the establishment of fast and sensitive technique of NASBA-ELISA toward diagnosing tuberculosis. This method is an isothermal amplification method based on RNA targets, and for this reason, it is able to assess the viability of the microorganism 14,15. The choice of EF-Tu mRNA as the target molecule in this study was a step toward achieving the goal (checking the viability) 16. This mRNA is found in all *M. tuberculosis* species abundantly, and consists 50% of cellular mRNA 16. EF-Tu protein is one of the important factors in translation process in prokaryotic cells and its expression is very necessary for the life of a cell 20.

This technique has an extraordinary precision, which is because of the enzyme activity of RNA polymerase (each pattern string containing T7 promoter is transcribed 100 to 1000 times). The combination of this technique with enzyme linked immunosorbent assay doubles the precision and specificity of the technique. NASBA-ELISA technique is consisted of 3 stages 21; in the first stage, RNA would be amplified by NASBA and simultaneously RNA amplicons labeled by digoxigenin (Dig-11-UTP). Then RNA-DNA hybrids would be formed. In the third stage, heteroduplex nucleic acid sequences will be detected by immunochemical assay.

With regard to the results achieved, the agarose gel electrophoresis due to its very low sensitivity and specificity cannot be used to identify the products of NASBA, but by entrance of a specific probe in ELISA system, the specificity of the technique increases incredibly.

In this study, not only EF-Tu mRNA of *M. tuberculosis* using DIG-Labeling NASBA technique could be amplified, but also the amplified products could be labeled. The whole process took 90 *min*. The degree of amplification was so high that the amplified fragment was well detectable on agarose gel. But the whole NASBA process takes place in 38 degrees and this is the reason it does not need a thermocycler, and the existence of non-specific amplification will be unavoidable. For this reason, ELISA system was used to identify the labeled NASBA products. In this system, by using a very specific oligonucleotide which was labeled with biotin, the amplified fragment in NASBA product could be easily identified. Many methods have been reported for detecting NASBA products like standard agarose gel electrophoresis 22, electrochemiluminescence-based techniques 23, the Enzyme-Linked Gel Assay (ELGA) 24 and real-time format by molecular beacons 25, but these methods either require advanced devices or do not have a high degree of precision. The ELISA system used in this study has a high degree of precision and specificity and eliminated the need for advanced devices. According to the results, 17.5 *pg* of RNA of *M. tuberculosis* present in 25 *μl* reaction mixture could be identified by NASBA-ELISA technique.

The important point is that contrary to other studies which used three T7 RNA polymerase, reverse transcriptase, RNase H enzymes in NASBA reaction 21–25, the same results could be reached with only two enzymes and omitting RNase H enzyme (Mentioned in the results sections) and this will have a significant role in lowering the costs and ease of work. Most reverse transcriptase enzymes have the property of RNase H and this property is such that it can replace RNase H enzyme 26.

The purpose from choosing EF-TumRNA in this study was to detect the viability of the microorganism besides identifying it. Till now, there is no report for detection of EF-Tu mRNA of *M. tuberculosis* with NASBA-ELISA method. To make sure that EF-Tu mRNA is only identifiable in living *M. tuberculosis*, NASBA-ELISA technique was tested on a number of dead bacteria in high temperature, and no amplification was observed in the samples with NASBA technique. In contrast, DNA of these dead bacteria was present in the samples proliferated easily with PCR. This result sheds light on two things:

First, NASBA is only effective on RNA and cannot amplify the DNA, and this is the basic problem in identifying prokaryotes, because PT-PCR which is the common way to identify RNA can cause the amplification of the DNA in the sample. Second, EF-Tu mRNA can be an identifying factor to detect the viability of the microorganisms.

## Conclusion

The NASBA-ELISA system offers several advantages in terms of sensitivity (97%), rapidity (only 4 *hr*) and simplicity (no need for thermo-cycler device) for detection of viable *M. tuberculosis*. Furthermore, due to its simplicity and high sensitivity feature, it could be used in limited access laboratories in a cost-effective manner. However, to make this technique common in medical centers, more time and more advertising are required, but it is hoped that the results of this project could be a step toward eliminating the problems of the diagnosis methods of tuberculosis in developing countries.
